# Association between thymidine kinase 1 and clinicopathological high-risk features in breast cancer

**DOI:** 10.1097/MD.0000000000050437

**Published:** 2026-08-28

**Authors:** Yanze Liu, Jiaqi Liu

**Affiliations:** a Department of Breast and Thyroid Surgery, Zibo Central Hospital, Zibo, Shandong, China.

**Keywords:** biomarker, breast cancer, high-risk factors, neoadjuvant chemotherapy, risk stratification, thymidine kinase 1

## Abstract

Thymidine kinase 1 (TK1) is a cell-cycle-regulated enzyme involved in DNA synthesis and is closely linked to tumor cell proliferation. This study investigated whether serum TK1 concentration is associated with clinicopathological high-risk features in breast cancer (BC) and whether TK1 changes after neoadjuvant chemotherapy (NACT). This retrospective single center cohort included 410 patients with invasive BC. Serum TK1 was analyzed according to age, lymph node metastasis (LNM), histological grade, Ki-67, circulating tumor cell (CTC) status, tumor size, and BC subtype. Subtypes were analyzed as Luminal A, Luminal B, and a human epidermal growth factor receptor 2 (HER2)-positive/triple-negative breast cancer (TNBC) high-risk group (25 HER2-positive and 35 TNBC cases). Multivariable linear regression included age, LNM, histological grade, Ki-67, CTC status, and subtype. Normality, sensitivity, and nonparametric analyses were performed. In the full-cohort, serum TK1 was higher in patients aged <35 years, in patients with LNM, in grade II/III tumors, and in tumors with Ki-67 > 30%. The univariable difference among the 3 subtype groups was not statistically significant (ANOVA *P* = .239); however, after adjustment, the HER2-positive/TNBC group had higher serum TK1 than Luminal A tumors (*B* = 0.197, 95% CI 0.103–0.291, *P* < .001). CTC status was not independently associated with TK1 (*B* = 0.020, 95% CI −0.081–0.122, *P* = .693). The full-cohort sensitivity model excluding CTC yielded consistent results. In the NACT subgroup, serum TK1 decreased after treatment overall and in both complete response and partial response subgroups. Serum TK1 was independently associated with younger age, LNM, higher histological grade, Ki-67, and the HER2-positive/TNBC high-risk phenotype. Serum TK1 also decreased after NACT. Because survival, recurrence-free survival, and disease-free survival were not evaluated, these findings support TK1 as a proliferation-related biomarker for risk stratification and treatment response monitoring, but not as definitive evidence of long-term outcome prediction.

## Abstract 2

This study evaluates the association between serum thymidine kinase 1 (TK1) concentration and clinicopathological high-risk features in breast cancer. In this retrospective cohort of 410 patients, serum TK1 was independently associated with younger age, lymph node metastasis, higher histological grade, Ki-67, and the human epidermal growth factor receptor 2-positive/triple-negative breast cancer high-risk phenotype. In 47 patients receiving neoadjuvant chemotherapy, serum TK1 decreased after treatment. Because survival and recurrence outcomes were not analyzed, TK1 is interpreted as a proliferation-related risk stratification and treatment monitoring biomarker rather than as a validated marker of long-term outcome.

## 1. Introduction

Breast cancer (BC) is one of the most prevalent malignancies worldwide and is characterized by marked heterogeneity in clinical presentation, molecular phenotype, treatment sensitivity, and outcome.^[1]^ Although early detection and systemic therapy have improved patient survival, clinicians still need accessible biomarkers that can complement conventional pathological assessment and reflect tumor biology during treatment.

This need is particularly relevant because patients with similar tumor size, nodal status, and receptor profile may have different proliferative activity and treatment responses. Biomarkers that are reproducible, minimally invasive, and dynamically responsive to therapy may refine risk stratification when interpreted alongside established clinicopathological factors.

BC subtype remains central to clinical decision-making. Hormone receptor (HR)-positive/human epidermal growth factor receptor 2 (HER2)-negative, HER2-positive, and triple-negative breast cancer (TNBC) differ substantially in biological behavior, therapeutic options, and response to neoadjuvant chemotherapy (NACT).^[2,3]^ TNBC is generally associated with high proliferative activity and limited targeted treatment options, whereas HER2-positive disease is biologically aggressive but responsive to HER2-directed therapy. HR-positive/HER2-negative disease is more strongly influenced by endocrine sensitivity and often shows a distinct response pattern to systemic therapy.

Thymidine kinase 1 (TK1) is a cytosolic enzyme in the thymidine salvage pathway. Its activity increases during S-phase and reflects DNA synthesis and cellular proliferation.^[4–8]^ Experimental and clinical studies have linked TK1 to tumor growth, cell-cycle activity, and treatment response across several malignancies.^[4,7]^ In BC, serum or tissue TK1 has been investigated as a proliferation-related marker, but its relationship with routinely used high-risk factors such as young age, lymph node metastasis (LNM), histological grade, Ki-67, molecular subtype, and circulating tumor cell (CTC) status remains incompletely defined.^[5,8]^

NACT provides an additional setting in which TK1 may be clinically informative. Because serum TK1 can be measured repeatedly, it may capture treatment-related changes in proliferative tumor cell activity. Previous studies have evaluated serum TK1 kinetics during neoadjuvant or systemic therapy, but results have varied by tumor subtype, treatment regimen, assay method, and sampling time point.^[5,9,10]^

The present study therefore evaluated whether serum TK1 concentration is associated with multiple clinicopathological high-risk features in a real-world BC cohort and whether these associations remain after multivariable adjustment. We also examined paired pre- and post-NACT TK1 changes in patients who received neoadjuvant treatment. The aim was to assess TK1 as a proliferation-related marker for risk stratification and treatment monitoring, without assuming definitive prognostic value in the absence of survival data.

## 2. Materials and methods

### 2.1. Study design and patient population

This was a retrospective, single-center cohort study conducted at Zibo Central Hospital. Consecutive eligible patients with invasive BC diagnosed from January 1, 2021, to December 31, 2024, were reviewed. A total of 410 patients met the inclusion criteria and were included in the analytic cohort. All patients were treated according to the NCCN Clinical Practice Guidelines in Oncology: BC (Version 6.2024) and CSCO BC Diagnosis and Treatment Guidelines 2024.^[3,11]^ The study was approved by the Institutional Review Board of Zibo Central Hospital (Approval Number: 2023 Research No. 100) and was conducted in accordance with the Declaration of Helsinki. Written informed consent was obtained for the use of anonymized clinical data and tissue/blood samples.

Among the 410 patients, 47 underwent NACT and were included in the paired analysis of serum TK1 concentration before and after treatment. Inclusion criteria were as follows:

1. Diagnosis of invasive BC confirmed by histopathology.
2. Availability of complete clinical and pathological data, including tumor subtype, age, Ki-67, lymph node status, and histological grade.
3. Availability of tumor tissue samples before and after chemotherapy (for the 47 patients who received NACT).
4. All enrolled patients had no distant metastases.

Exclusion criteria included:

1. Noninvasive BCs (e.g., ductal carcinoma in situ).
2. Incomplete clinical data.
3. Prior treatment with systemic chemotherapy or radiation therapy before diagnosis.
4. Lack of consent for tissue collection.

### 2.2. Study size, missing data, and bias control

**Figure 1.**
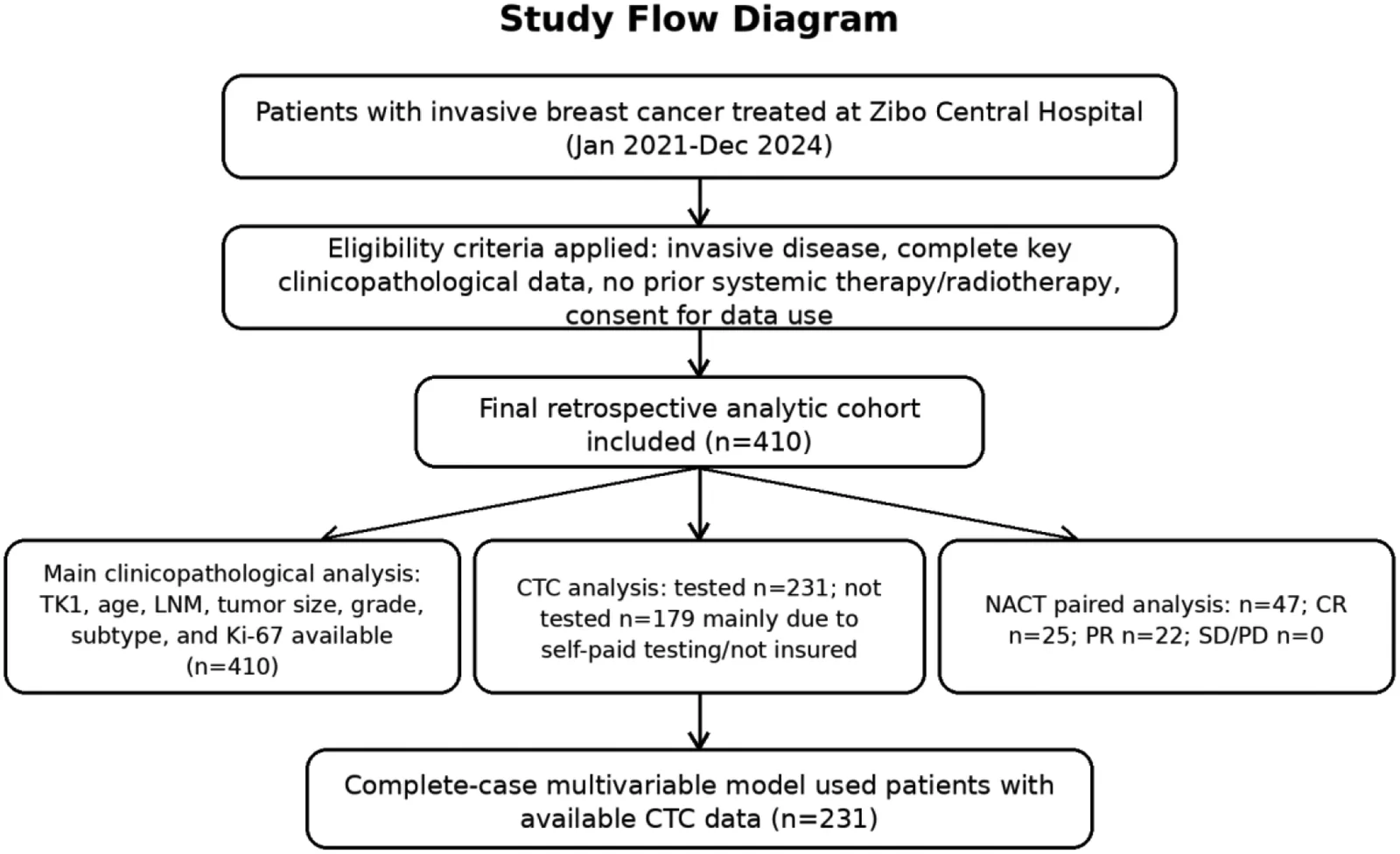
Study flow diagram. The diagram summarizes the analytic cohort, the CTC-tested subgroup, and the NACT subgroup used in this retrospective study. CTC = circulating tumor cell, NACT = neoadjuvant chemotherapy.

The study size was determined by the number of consecutive eligible patients available during the prespecified study period; no formal a priori sample-size calculation was performed. Key clinicopathological variables required for the primary analysis, including TK1, age, LNM, tumor size, histological grade, Ki-67, and subtype, were complete for all 410 included patients. CTC data were available for 231 patients; the remaining 179 patients did not undergo CTC testing. Therefore, the primary multivariable model including CTC used complete cases, and a full-cohort sensitivity model excluding CTC was performed. The study flow diagram is shown in Figure 1.

To reduce information and measurement bias, TK1 testing was performed using the same platform and reagent lot, and all procedures were completed by a trained technician according to standardized protocols. Multivariable regression and sensitivity analyses were used to address confounding and assess the stability of the findings.

### 2.3. BC subtype classification

BC subtypes were assigned using ER, progesterone receptor (PR), HER2, and Ki-67 results from routine pathological reports. In the analytic dataset, the subtype variable was coded as follows: code 1, Luminal A; code 2, Luminal B; and code 3, a high-risk group including HER2-positive BC and TNBC. The code 3 group included 25 HER2-positive cases and 35 TNBC cases.

- Luminal A was defined as HR-positive/HER2-negative disease with a low proliferative profile according to institutional pathological criteria.
- Luminal B was defined as HR-positive disease with a higher proliferative profile. HER2-positive cases were not double-counted in the luminal categories for the primary analysis.
- HER2-positive BC was defined as HER2 immunohistochemistry (IHC) 3 + or HER2 gene amplification by fluorescence in situ hybridization (FISH), regardless of ER/PR status. TNBC was defined as ER-negative, PR-negative, and HER2-negative disease.

For overlapping HR-positive/HER2-positive tumors, the prespecified rule was to classify them as HER2-positive rather than HR-positive to avoid double-counting and to reflect the dominant treatment strategy. For standard intrinsic-like reporting, the available subtype data permit reporting of HR-positive/HER2-negative luminal tumors, HER2-positive tumors regardless of HR status, and TNBC, but do not support a fully separated 4-category analysis of HR-positive/HER2-negative, HR-positive/HER2-positive, HR-negative/HER2-positive, and TNBC.

### 2.4. NACT protocol

The 47 patients who underwent NACT were treated according to institutional protocols based on tumor subtype. All treatment options referred to the NCCN Clinical Practice Guidelines in Oncology: BC (Version 6.2024) and CSCO BC Diagnosis and Treatment Guidelines 2024.^[3,11]^

HR-positive/HER2-negative BC and TNBC: patients received the TAC regimen consisting of albumin-bound paclitaxel 260 mg/m^2^, pirarubicin 50 mg/m^2^, and cyclophosphamide 500 mg/m^2^, administered intravenously every 21 days for 6 cycles. No patient in this cohort received epirubicin.

HER2-positive BC: patients received the TCbHP regimen consisting of albumin-bound paclitaxel 260 mg/m^2^, carboplatin AUC 6, trastuzumab (8 mg/kg loading dose followed by 6 mg/kg maintenance), and pertuzumab (840 mg loading dose followed by 420 mg maintenance), administered intravenously every 21 days for 6 cycles.

Treatment response after NACT was assessed by post-treatment clinical and radiological evaluation and was supplemented by postoperative pathological review when available. Post-NACT blood sampling for TK1 was performed after completion of the planned NACT regimen during the preoperative reassessment period and before initiation of adjuvant endocrine therapy. The retrospective records documented complete response (CR) and PR rather than uniformly standardized pCR status; therefore, the term CR was used conservatively and was not interpreted as a uniform pathological CR endpoint. No stable disease (SD) or progressive disease (PD) cases were present in the NACT subgroup. Because the NACT subgroup was small and had no untreated control group, subtype-adjusted or pCR-predictive modeling was not performed to avoid unstable estimates.

1. CR: no detectable residual lesion by the available post-treatment assessment.
2. Partial response (PR): a decrease in tumor size by at least 50% without complete resolution.
3. SD: no significant change in tumor size; no SD cases were present in this cohort.
4. PD: increase in tumor size or new lesions; no PD cases were present in this cohort.

### 2.5. Measurement of serum TK1

1. Sample collection: Peripheral venous blood (5 mL) was collected on the second morning after admission after overnight fasting. Samples with moderate to severe hemolysis or lipemia were rejected. Serum was separated within 3 hours after blood coagulation by centrifugation at 3000 rpm for 10 minutes and stored at −20°C until testing.
2. Detection method: Serum TK1 was measured using the SSTKBIO TK1 cell-cycle assay kit according to the manufacturer’s instructions. Briefly, serum samples were spotted onto a nitrocellulose membrane, dried, blocked, incubated with a primary antibody against TK1, and then incubated with an HRP-conjugated secondary antibody. Chemiluminescent substrate was added, and signal intensity was detected using an imaging system. TK1 concentration was determined from a standard curve.

Analytical performance and quality control: Serum TK1 concentrations were reported in pmol/L. Analytical performance characteristics, including limit of detection (LoD), limit of quantification (LoQ), and linear range, were based on the manufacturer’s validation data for the SSTKBIO TK1 cell cycle assay kit. The enhanced chemiluminescence dot-blot system has a reported analytical sensitivity of 10^-12 μg/L. All measured samples fell within the validated linear range of the standard curve. According to manufacturer-confirmed quality control data, the intra-assay coefficient of variation was <10% and the inter-assay coefficient of variation was <15%. Each sample was measured in duplicate, and the mean value was used for analysis. To minimize batch effects, all serum samples were tested on the same platform with the same lot of TK1 assay kits by one trained technician following standardized operating procedures.

### 2.6. Measurement of CTC

1. Sample Collection: Peripheral venous blood (7.5 mL) was collected on the second morning of admission after fasting. Samples with moderate to severe hemolysis or lipemia were rejected. After sample collection, the sample collection tubes were stored at 15–30°C and processed for testing within 24 hours.
2. Detection Method: CTC testing was offered to all eligible participants in this cohort. A total of 231 patients consented to CTC testing and completed the testing procedure, whereas the remaining patients declined testing mainly because the assay was a self-paid examination and was not covered by standard medical insurance during the study period. CTCs were detected using an immunostaining-fluorescence in situ hybridization platform. Tumor cell identification was performed using FISH with chromosome enumeration probes for chromosomes 7, 8, and 17 (CEP7, CEP8, and CEP17). CTC positivity was defined as the detection of ≥1 CTC per 7.5 mL of peripheral blood, in accordance with the manufacturer-validated protocol and established literature thresholds. All slides were scanned using an automated fluorescence microscopy system and independently interpreted by 2 trained technicians who were blinded to the clinical outcomes. To evaluate possible selection bias, baseline characteristics of CTC-tested and non-tested patients were compared and are reported in the Results.

### 2.7. Statistical analysis

All statistical analyses were performed using the study dataset. Continuous variables were summarized as mean ± standard deviation and, when appropriate, median with interquartile range. Distributional normality of TK1 was evaluated using histograms, Q-Q plots, skewness/kurtosis, and the Shapiro–Wilk test. Between-group comparisons were performed using Welch’s *t* test or one-way ANOVA for parametric analyses and Mann-Whitney *U* or Kruskal-Wallis tests for nonparametric sensitivity analyses. Paired comparisons of pre- and post-NACT TK1 were performed using paired tests, and effect sizes with 95% confidence intervals were reported when applicable. Multiple testing was addressed using the Benjamini-Hochberg false-discovery-rate method.

Multivariable linear regression was used to evaluate independent associations between serum TK1 concentration and age, LNM, histological grade, Ki-67, CTC status, and subtype. Subtype was modeled categorically, with Luminal A as the reference category. Because CTC data were available for 231 patients, the primary multivariable model including CTC was performed as a complete-case analysis. A full-cohort sensitivity model excluding CTC was performed in all 410 patients. Model diagnostics included R^2^, adjusted R^2^, *F*-statistic, residual normality testing, heteroscedasticity testing, and variance inflation factor assessment. Heteroscedasticity-heteroscedasticity-robust standard errors were reported.

### 2.8. Ethical considerations

The study was conducted in accordance with the ethical principles outlined in the Declaration of Helsinki. All patient data were anonymized, and patient confidentiality was strictly maintained. The study protocol was approved by the Institutional Review Board of Zibo Central Hospital (Approval Number: 2023 Research No. 100). We confirm that all experiments were performed in accordance with relevant guidelines and regulations. The transparency in data reporting and conflict of interest disclosures in accordance with journal requirements.

### 2.9. Informed consent

Informed consent was obtained from all individual participants included in the study. The patients signed an informed consent form for their data to be used anonymously for research purposes. Patient consent was obtained in compliance with ethical guidelines.

### 2.10. Data availability

The datasets analyzed during the current study are available from the corresponding author on reasonable request, subject to institutional and ethical restrictions on patient-level clinical data.

## 3. Results

### 3.1. Patient demographics and baseline characteristics

A total of 410 patients were included. In the full cohort, TK1 was higher in patients aged <5 years than in those aged ≥35 years (5.184 ± 0.623 vs 4.451 ± 0.708, *P* < .001). TK1 was also higher in patients with LNM (5.181 ± 0.494 vs 4.071 ± 0.478, *P* < .001), grade II/III tumors (4.582 ± 0.643 vs 3.000 ± 0.641, *P* < .001), and Ki-67 > 30% (4.554 ± 0.732 vs 4.391 ± 0.705, *P* = .034). Across the 3 recorded subtype groups, TK1 did not differ significantly in the univariable analysis (ANOVA *P* = .239). CTC-positive and CTC-negative patients had similar serum TK1 concentrations (4.459 ± 0.715 vs 4.533 ± 0.750, *P* = .579). In tumor size-stratified analyses, LNM remained associated with higher TK1 in both tumors <2 cm and tumors >2 cm. The demographic and baseline characteristics of the patients are shown in Table 1.

**Table 1:** Patient demographics and baseline characteristics. Are TK1 concentrations expressed in pmol/L.

Clinical feature	No. of cases	TK1, pmol/L (mean ± SD)	*P*-value
Age	Total = 410		
<35 yr	30	5.184 ± 0.623	<.001
≥35 yr	380	4.451 ± 0.708	
Axillary LNM	Total = 410		
Yes	160	5.181 ± 0.494	<.001
No	250	4.071 ± 0.478	
Histological grade	Total = 410		
Grade I	20	3.000 ± 0.641	<.001
Grade II/III	390	4.582 ± 0.643	
Ki-67	Total = 410		
>30%	285	4.554 ± 0.732	.034
≤30%	125	4.391 ± 0.705	
BC subtype	Total = 410		
Luminal A	296	4.525 ± 0.687	.239
Luminal B	54	4.349 ± 0.794	
HER2-positive/TNBC	60	4.544 ± 0.844	
CTCs	Total = 231		
≥1 CTC	35	4.459 ± 0.715	.579
0 CTC	196	4.533 ± 0.750	
Tumor < 2 cm stratified by LNM	Total = 208		
Tumor < 2 cm with LNM	78	5.374 ± 0.404	<.001
Tumor < 2 cm without LNM	130	4.071 ± 0.452	
Tumor > 2 cm stratified by LNM	Total = 202		
Tumor > 2 cm with LNM	82	4.998 ± 0.504	<.001
Tumor > 2 cm without LNM	120	4.071 ± 0.507	

*P*-values were obtained using Welch’s *t* test for 2-group comparisons and ANOVA for the 3-subtype comparison.

BC = breast cancer, CTC = circulating tumor cell, HR = hormone receptor, LNM = lymph node metastasis, TK1 = thymidine kinase 1, TNBC = triple-negative breast cancer, HER2 = human epidermal growth factor receptor 2.

### 3.2. Serum TK1 before and after NACT

Among the 47 patients who underwent NACT, TK1 decreased after treatment overall and within both response categories (Table 2). The overall mean TK1 reduction was 0.976 pmol/L (approximate 95% CI 0.761–1.191; Hedges g_av = 1.84; *P* < .001). In the CR group (n = 25), TK1 decreased from 4.418 ± 0.469 to 3.476 ± 0.453 pmol/L (mean reduction 0.942, approximate 95% CI 0.680–1.204; Hedges g_av = 2.01; *P* < .001). In the PR group (n = 22), TK1 decreased from 4.902 ± 0.474 to 3.920 ± 0.496 pmol/L (mean reduction 0.982, approximate 95% CI 0.687–1.277; Hedges g_av = 1.99; *P* < .001). No SD or PD cases were recorded. These results should be interpreted as paired biomarker changes after treatment rather than evidence that TK1 independently predicts pCR or survival.

**Table 2:** TK1 concentrations before and after NACT in BC patients. Are TK1 concentrations expressed in pmol/L.

Response	n	Pre-NACT TK1, pmol/L (mean ± SD)	Post-NACT TK1, pmol/L (mean ± SD)	Mean reduction, pmol/L	95% CI for reduction	Hedges g_av	*P*-value
Overall	47	4.681 ± 0.567	3.705 ± 0.481	0.976	0.761 to 1.191	1.84	<.001
CR group	25	4.418 ± 0.469	3.476 ± 0.453	0.942	0.680–1.204	2.01	<.001
PR group	22	4.902 ± 0.474	3.920 ± 0.496	0.982	0.687–1.277	1.99	<.001

The 95% CIs for TK1 reduction were approximated from the available summary statistics; exact paired CIs require individual paired pre-/post-TK1 concentrations.

Treatment regimens: all treatment options were based on CSCO Breast Cancer Diagnosis and Treatment Guidelines 2024 and NCCN Clinical Practice Guidelines in Oncology: Breast Cancer (Version 6.2024). HR-positive/HER2-negative breast cancer and TNBC patients received albumin-bound paclitaxel, pirarubicin, and cyclophosphamide every 21 days for 6 cycles. No patient received epirubicin. HER2-positive breast cancer patients received albumin-bound paclitaxel, carboplatin, trastuzumab, and pertuzumab every 21 days for 6 cycles. Endocrine therapy was not administered concurrently with NACT. Anti-HER2 therapy was administered concurrently as part of the standard TCbHP regimen and is acknowledged as a potential contributor to TK1 changes in HER2-positive patients.

CR: no detectable residual lesion by the available post-treatment assessment.

PR: a decrease in tumor size by at least 50% but not complete resolution.

NACT = neoadjuvant chemotherapy, CR = complete response, PR = partial response, TK1 = thymidine kinase 1.

### 3.3. Multivariable linear regression analysis

In the complete-case multivariable model including age, LNM, histological grade, Ki-67, CTC status, and subtype (see Table 3), the model showed high explanatory performance (*R*^2^ = 0.845, adjusted *R*^2^ = 0.840; F = 173.5, *P* < .001; RMSE = 0.292). Model diagnostics are presented in Table 4 (see Table 4). Age, LNM, and grade remained statistically significant after adjustment. Specifically, younger age was associated with higher TK1 (*B* = −0.009 per year, 95% CI −0.013–−0.006, *P* < .001), LNM was independently associated with higher TK1 (*B* = 1.153, 95% CI 1.071–1.235, *P* < .001), and grade II/III tumors had higher TK1 than grade I tumors (*B* = 1.666, 95% CI 1.442–1.889, *P* < .001). Ki-67 also remained significant (*B* = 0.002 per 1%, *P* = .002). CTC status was not significant (*B* = 0.020, 95% CI −0.081–0.122, *P* = .693). Compared with Luminal A tumors, the HER2-positive/TNBC group showed higher adjusted TK1 (*B* = 0.197, 95% CI 0.103–0.291, *P* < .001), whereas Luminal B did not differ significantly from Luminal A. In the full-cohort sensitivity model excluding CTC (n = 410) (see Table 5), the direction and significance of the main associations were unchanged, supporting the robustness of the complete-case findings.

**Table 3:** Multivariable linear regression model for serum TK1 concentration (complete-case model including CTC, n = 231).

Variable	Coefficient B	Robust SE	95% CI	t	*P*-value	VIF
Intercept	2.866	0.163	2.545–3.186	17.61	<.001	
Age (per yr)	−0.009	0.002	−0.013–−0.006	−5.54	<.001	1.04
LNM (yes vs no)	1.153	0.041	1.071–1.235	27.84	<.001	1.01
Grade II/III vs I	1.666	0.113	1.442–1.889	14.70	<.001	1.02
Ki-67 (per 1%)	0.002	0.001	0.001–0.003	3.21	.002	1.01
CTC positive	0.020	0.052	−0.081–0.122	0.40	.693	1.02
Luminal B vs Luminal A	−0.071	0.073	−0.215–0.073	−0.97	.333	1.08
HER2+/TNBC vs Luminal A	0.197	0.048	0.103–0.291	4.13	<.001	1.05

BC = breast cancer, CTC = circulating tumor cell, LNM = lymph node metastasis. The model used complete cases with available CTC data (n = 231). HC3 heteroscedasticity-robust standard errors are reported because heteroscedasticity was detected.

**Table 4:** Multivariable regression model diagnostics.

Diagnostic	Value
No. of observations	231
R^2^/ adjusted R^2^	0.845/ 0.840
*F*-statistic (model *P*-value)	173.5 (*P* < .001)
RMSE	0.292
AIC/ BIC	103.2/ 130.8
Durbin-Watson	1.441
Breusch-Pagan test	*χ*^2^ = 28.27, *P* < .001
Residual normality (Shapiro–Wilk)	W = 0.987, *P* = .036
Maximum VIF	1.08

**Table 5:** Full-cohort sensitivity multivariable regression model excluding CTC (n = 410).

Variable	Coefficient B	Robust SE	95% CI	Statistic	*P*-value
Intercept	2.901	0.109	2.688–3.114	26.70	<.001
Age (per yr)	−0.008	0.001	−0.011–−0.006	-6.32	<.001
LNM (yes vs no)	1.116	0.033	1.052–1.180	34.20	<.001
Grade II/III vs I	1.606	0.075	1.458–1.753	21.34	<.001
Ki-67 (per 1%)	0.002	0.001	0.001–0.003	3.34	.001
Luminal B vs Luminal A	−0.092	0.053	−0.196–0.011	−1.76	.079
HER2+/TNBC vs Luminal A	0.143	0.041	0.062–0.224	3.48	.001

This model was fitted to assess whether the complete-case restriction caused by missing CTC data materially influenced the main findings. *R*^2^ = 0.818, adjusted *R*^2^ = 0.815, and the main associations were consistent with the complete-case model including CTC.

### 3.4. CTC-tested subgroup, normality checks, and sensitivity analyses

**Figure 2.**
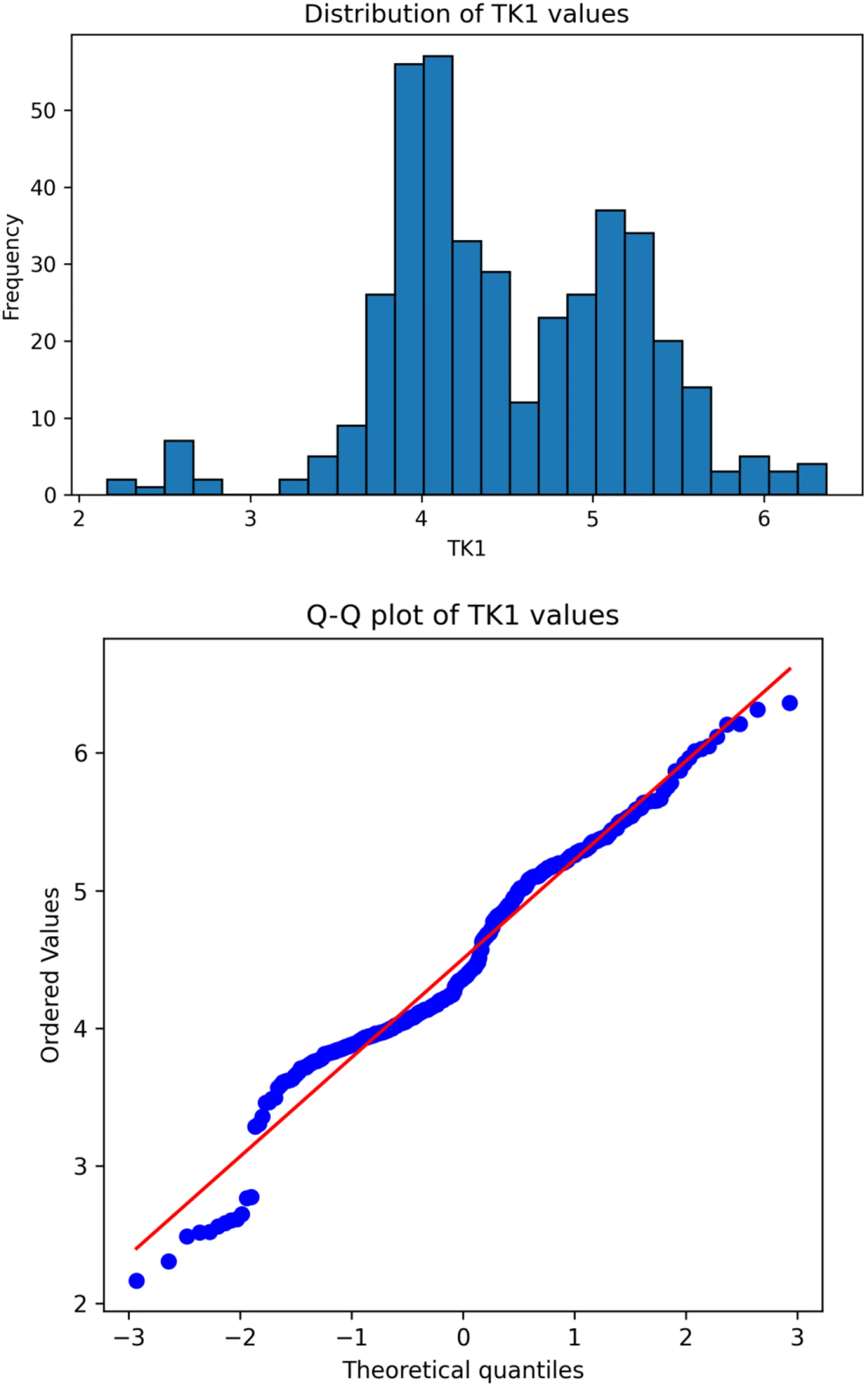
Distribution plots for TK1. Upper panel: histogram of TK1 concentrations; lower panel: (Q-Q) plot of TK1 concentrations. TK1 = thymidine kinase 1.

CTC testing was completed in 231 patients. The remaining 179 patients declined testing mainly because the assay was a self-paid examination and was not covered by standard medical insurance during the study period. To evaluate potential selection bias, we compared measured baseline characteristics between CTC-tested and non-tested patients. No statistically significant differences were observed in age, tumor size, histological grade, or molecular subtype distribution (all *P* > .05; Table 6), suggesting that missing CTC data were unlikely to introduce systematic selection bias into the present analysis. TK1 showed a statistically significant Shapiro–Wilk test because of the large sample size, but skewness and kurtosis were small. Log transformation did not improve normality. Nonparametric sensitivity analyses were therefore performed and reported in Table 7, Table 8, and Figure 2.

**Table 6:** Comparison of patients with and without CTC testing.

Variable	CTC-tested (n = 231)	Not CTC-tested (n = 179)	*P*-value
Age, yr	55.433 ± 14.459	55.760 ± 14.747	.822
TK1, pmol/L	4.522 ± 0.744	4.482 ± 0.706	.574
Ki-67, %	48.437 ± 30.249	51.469 ± 29.837	.311
LNM-positive	92 (39.8%)	68 (38.0%)	.782
Tumor > 2 cm	112 (48.5%)	90 (50.3%)	.794
Grade II/III	220 (95.2%)	170 (95.0%)	1.000
Subtype distribution	Luminal A 165 (71.4%); Luminal B 27 (11.7%); HER2+/TNBC 39 (16.9%)	Luminal A 131 (73.2%); Luminal B 27 (15.1%); HER2+/TNBC 21 (11.7%)	.252

**Table 7:** Distribution checks and sensitivity analyses.

Distribution check	Result
Overall TK1, pmol/L	mean ± SD 4.504 ± 0.727; median 4.367 (IQR 3.987–5.108)
Shapiro–Wilk test for TK1	W = 0.968, *P* < .001
Skewness/ kurtosis	–0.116/ 0.133
Log transformation	Shapiro–Wilk W = 0.937, *P* < .001; no improvement in normality
Multiplicity correction	Benjamini-Hochberg false-discovery-rate correction was applied across the 6 primary univariable comparisons.

**Table 8:** Nonparametric sensitivity analyses and multiplicity correction.

Analysis	Parametric *P*	Nonparametric *P*	BH-FDR adjusted *P*
Age < 35 vs ≥ 35	<.001	<.001	<.001
LNM yes vs no	<.001	<.001	<.001
Grade I vs II/III	<.001	<.001	<.001
Ki-67 > 30% vs ≤ 30%	.034	.013	.019
Subtype 3 vs subtypes 1/2	.690	.355	.426
CTC positive vs negative	.579	.759	.759

Parametric analyses used Welch’s *t* test for 2-group comparisons and one-way ANOVA for the 3-subtype comparison; nonparametric sensitivity analyses used Mann-Whitney U or Kruskal-Wallis tests. BH-FDR, Benjamini-Hochberg false-discovery-rate correction.

## 4. Discussion

The present cohort provides several clinically meaningful observations regarding serum TK1 in BC. First, TK1 was higher in patients with established high-risk clinicopathological features, including younger age, LNM, higher histological grade, and high Ki-67 expression. Second, crude subtype comparisons showed only modest differences among Luminal A, Luminal B, and the HER2-positive/TNBC high-risk group; however, after adjustment for age, LNM, grade, Ki-67, and CTC status, the HER2-positive/TNBC group showed higher TK1 than Luminal A disease. Third, TK1 decreased substantially after NACT in both CR and partial response groups. Taken together, these findings indicate that serum TK1 is best interpreted as a proliferation-related and treatment-dynamic biomarker rather than as a stand-alone prognostic marker. Because recurrence, disease-free survival, and overall survival were not available, the present data support an association with high-risk biology but do not establish independent prognostic validity.

### 4.1. Biological interpretation of serum TK1 in BC

TK1 is a cytosolic enzyme in the thymidine salvage pathway and is closely linked to DNA synthesis, S-phase progression, and cellular proliferation.^[12]^ This biological role provides a plausible explanation for why circulating TK1 may increase in tumors with greater proliferative activity. Pan-cancer analyses also support the association between serum TK1 concentration and proliferation-related oncogenic programs.^[4]^ In BC specifically, tissue-based TK1 IHC has been explored as a tool for risk stratification, although the relationship between tissue TK1, serum TK1, and clinical outcomes remains incompletely defined.^[8]^ The current study adds to this literature by focusing on serum TK1 in a real-world BC cohort and by linking it to routine clinicopathological factors that are commonly available in clinical practice.

A key point is that serum TK1 should not be viewed as a replacement for pathological evaluation. Rather, it may capture a systemic signal of tumor proliferation and treatment-related tumor cell turnover. This distinction is important because tissue markers such as histological grade and Ki-67 provide information from sampled tumor tissue, whereas serum TK1 may reflect a broader biological process, including proliferative burden and treatment-induced changes. The consistent direction of association across age, nodal status, grade, and Ki-67 in the present cohort supports the interpretation of TK1 as a complementary marker of tumor biological activity.

### 4.2. Association between TK1 and clinicopathological high-risk features

In this study, patients younger than 35 years had higher serum TK1 concentrations than older patients (5.184 ± 0.623 vs 4.451 ± 0.708 pmol/L, *P* < .001). Younger age at BC diagnosis is often associated with biologically aggressive disease, higher grade, higher proliferative indices, and a greater proportion of unfavorable molecular subtypes.^[13]^ Therefore, the association between younger age and TK1 is biologically credible. However, age itself should not be interpreted as the direct cause of increased TK1. Instead, TK1 may reflect the proliferative tumor phenotype that is more frequently encountered in younger patients.

The strongest and most clinically relevant association was observed for LNM. Patients with LNM had markedly higher TK1 than node-negative patients (5.181 ± 0.494 vs 4.071 ± 0.478 pmol/L, *P* < .001), and LNM remained independently associated with TK1 in the multivariable model (*B* = 1.153, 95% CI 1.071–1.235, *P* < .001). This finding was consistent even when tumor size was considered: among tumors <2 cm, TK1 was higher in node-positive than node-negative disease (5.374 ± 0.404 vs 4.071 ± 0.452 pmol/L, *P* < .001), and a similar pattern was observed among tumors >2 cm (4.998 ± 0.504 vs 4.071 ± 0.507 pmol/L, *P* < .001). These results suggest that TK1 is not simply a surrogate for primary tumor size. Instead, it may reflect a more invasive and biologically active phenotype associated with nodal spread.

Histological grade was also strongly associated with TK1. Grade II/III tumors showed substantially higher TK1 than grade I tumors (4.582 ± 0.643 vs 3.000 ± 0.641 pmol/L, *P* < .001), and this association remained robust after adjustment (*B* = 1.666, 95% CI 1.442–1.889, *P* < .001). Because histological grade integrates tubule formation, nuclear pleomorphism, and mitotic activity, its association with TK1 further supports the link between serum TK1 and proliferative tumor biology. The magnitude of the grade effect in this cohort also suggests that TK1 may be particularly informative in distinguishing indolent tumors from tumors with more active cellular turnover.

The relationship between Ki-67 and TK1 requires more nuanced interpretation. Patients with Ki-67 > 30% had higher TK1 than those with Ki-67 ≤ 30% (4.554 ± 0.732 vs 4.391 ± 0.705 pmol/L, *P* = .034), and Ki-67 remained independently associated with TK1 in the adjusted model. The International Ki-67 Working Group has emphasized that very low and very high Ki-67 values are more clinically interpretable than intermediate values, and the 30% threshold has practical relevance in risk assessment.^[14]^ The modest effect size observed here is therefore not unexpected. Ki-67 and TK1 assess related but non-identical biological dimensions: Ki-67 is a tissue-based nuclear proliferation index, whereas TK1 is a serum marker linked to DNA synthesis and cellular turnover. Their association supports biological coherence, but the 2 markers should be regarded as complementary rather than interchangeable.

### 4.3. TK1 across BC subtypes

Subtype classification in this study was based on routine postoperative pathological assessment of estrogen receptor (ER), PR, HER2, and Ki-67, using the St. Gallen surrogate framework. HR-positive BC was defined as ER and/or PR positivity (≥1%) with HER2 negativity (IHC 0/1+, or IHC 2+ with FISH non-amplification). HER2-positive BC was defined as HER2 positivity (IHC 3+, or IHC 2+with FISH amplification), irrespective of HR status. TNBC was defined as negativity for ER, PR, and HER2[2,15]. For overlapping HR+/HER2+ tumors, classification was guided by the dominant treatment strategy and biological behavior. Although these tumors express HRs, neoadjuvant systemic therapy is primarily driven by HER2-directed therapy combined with chemotherapy, whereas endocrine therapy is usually administered as subsequent adjuvant treatment. In addition, HR+/HER2+ tumors generally show greater proliferative activity than HR-positive/HER2-negative disease. Therefore, from the perspectives of treatment decision-making and biological aggressiveness, HR+/HER2+ cases were included in the HER2-positive/TNBC high-risk group for the exploratory subtype analysis.

The rationale for comparing the combined TNBC/HER2-positive group with HR-positive/HER2-negative disease was also related to the biological function of TK1. TNBC and HER2-positive BC, including HR+/HER2 + disease, are commonly characterized by higher proliferation, greater biological aggressiveness, and chemotherapy-centered neoadjuvant treatment strategies. Because TK1 reflects DNA synthesis and proliferative tumor cell turnover, its circulating concentration and treatment-related changes may be more comparable across these high-proliferation subtypes. By contrast, HR-positive/HER2-negative BC is more strongly influenced by endocrine sensitivity, and its neoadjuvant treatment selection and response evaluation logic differ substantially from those of TNBC and HER2-positive disease. Thus, this dichotomous grouping was used as a clinically pragmatic framework of high-proliferation/high-aggressiveness versus relatively endocrine-sensitive disease, rather than as a replacement for standard intrinsic-like classification.

Within this framework, subtype analysis showed a nuanced pattern. In the univariable comparison, TK1 did not differ significantly among Luminal A, Luminal B, and the HER2-positive/TNBC high-risk group (*P* = .239), indicating that subtype alone does not fully explain serum TK1 variation. Luminal tumors, particularly Luminal B tumors, may also display substantial proliferative heterogeneity. Conversely, serum TK1 concentrations in HER2-positive or TNBC tumors may be influenced by tumor burden, nodal status, treatment timing, and other biological factors.

In the CTC-adjusted multivariable model, the HER2-positive/TNBC group had higher TK1 than Luminal A tumors (*B* = 0.197, 95% CI 0.103–0.291, *P* < .001), and the full-cohort sensitivity model excluding CTC showed the same direction of association (*B* = 0.143, 95% CI 0.062–0.224, *P* = .001). This adjusted association is consistent with the high-proliferation phenotype of HER2-positive and triple-negative BCs and with the clinical practice of interpreting subtype together with grade, Ki-67, nodal status, and treatment sensitivity.^[15,16]^ Nevertheless, the combined HER2-positive/TNBC group included only 60 cases, comprising 25 HER2-positive and 35 TNBC cases. Therefore, the subtype result should be interpreted as exploratory. Future studies should prospectively evaluate HR-positive/HER2-negative, HR-positive/HER2-positive, HR-negative/HER2-positive, and TNBC as separate categories to determine whether TK1 has distinct biological and clinical implications across standard intrinsic-like subtypes.

### 4.4. Dynamic changes in TK1 after NACT

The NACT subgroup provides one of the most clinically relevant observations in this study. Among 47 patients who received NACT, TK1 decreased from 4.681 ± 0.567 to 3.705 ± 0.481 pmol/L, with a mean reduction of 0.976 pmol/L (95% CI 0.761–1.191, *P* < .001; Hedges g_av = 1.84). This large within-patient change indicates that serum TK1 is sensitive to systemic therapy and may reflect reduced proliferative activity or decreased viable tumor burden after treatment. The decrease was observed in both the CR group (mean reduction 0.942 pmol/L) and PR group (mean reduction 0.982 pmol/L), suggesting that TK1 may capture a general treatment effect rather than strictly distinguishing complete from partial response.

These results should be interpreted together with prior TK1 studies in BC. Bolayirli et al reported that serum TK1 activity changed after adjuvant chemotherapy in patients with solid tumors, including BC.^[17]^ Bagegni et al demonstrated that serum TK1 activity could serve as a pharmacodynamic marker of CDK4/6 inhibition in early-stage ER-positive BC.^[18]^ In the PROMIX neoadjuvant cohort, Matikas et al found that TK1 kinetics during NACT provided long-term prognostic information.^[10]^ In contrast, the PREDIX HER2 analysis by Zhu et al showed that serial serum TK1 activity in HER2-positive disease increased during neoadjuvant anti-HER2-based therapy and was not associated with pCR or time-to-event outcomes.^[5]^ These differences indicate that TK1 dynamics may depend on tumor subtype, treatment regimen, sampling time point, assay platform, and whether TK1 activity or concentration is measured.

The absence of SD or PD cases in the NACT subgroup limits the ability to evaluate whether TK1 can identify treatment resistance. In addition, response was recorded as CR or PR rather than uniformly documented pathological complete response (pCR) or residual cancer burden. This distinction is important because pCR, particularly ypT0/is ypN0 or ypT0 ypN0, has been repeatedly associated with improved long-term outcomes after neoadjuvant therapy, with the strongest prognostic relevance in TNBC and HER2-positive disease.^[19,20]^ Future TK1 studies should therefore incorporate predefined sampling points, standardized pCR or residual cancer burden assessment, and subtype-stratified models to determine whether TK1 changes add information beyond conventional response evaluation.

### 4.5. Interpretation of the CTC findings

CTC positivity was not associated with TK1 in the 231 patients who underwent CTC testing. Patients with ≥1 CTC had serum TK1 concentrations similar to those without detectable CTCs (4.459 ± 0.715 vs 4.533 ± 0.750 pmol/L, *P* = .579), and CTC positivity was not significant in the multivariable model. This negative result does not necessarily contradict the biological relevance of either marker. CTCs represent hematogenous tumor cell dissemination, whereas TK1 primarily reflects proliferation-related enzymatic activity and tumor cell turnover. These are related but distinct dimensions of tumor biology.

Recent reviews indicate that CTCs have clinical validity in BC, particularly in prognostication, but their interpretation depends heavily on disease stage, assay method, timing of blood collection, and positivity threshold.^[21]^ In localized or early BC, CTC counts are often low, and a threshold of ≥1 CTC/7.5 mL has frequently been used. Such low-count data may have limited ability to correlate with continuous serum biomarkers. Therefore, the lack of association between CTC status and TK1 in this cohort suggests that TK1 should not be considered a substitute for CTC testing; instead, TK1 and CTCs may provide different types of information.

### 4.6. Clinical implications

The practical value of TK1 lies in its potential integration with routine pathological variables rather than in isolated interpretation. In this cohort, TK1 was aligned with age-related risk, nodal metastasis, histological grade, Ki-67, and treatment response. If validated prospectively, serum TK1 could serve as an adjunctive marker for identifying patients with biologically active disease and for monitoring systemic treatment dynamics. Its blood-based nature makes serial assessment feasible, which is an advantage over tissue-only biomarkers. However, the present results should not be used to guide treatment escalation or de-escalation without prospective validation.

The most appropriate future direction is not simply to test whether TK1 is “high” or “low,” but to evaluate whether baseline TK1 and early TK1 kinetics improve risk models that already include age, tumor size, nodal status, grade, Ki-67, molecular subtype, and treatment regimen. Prospective studies should also determine assay-specific cutoffs, assess reproducibility across platforms, and evaluate whether TK1 adds independent information for pCR, recurrence-free survival, disease-free survival, and overall survival. Such studies would clarify whether TK1 is merely correlated with proliferation or whether it has actionable value in BC management.

### 4.7. Limitations

This study has several limitations. First, it was retrospective and conducted at a single center, which may introduce selection bias and restrict external validity. Second, survival outcomes, recurrence-free survival, disease-free survival, and overall survival were not available; therefore, the study cannot determine whether TK1 is independently prognostic for long-term outcomes. Third, although CTC testing was offered to all eligible patients, only 231 patients completed testing because the assay was self-paid and was not covered by standard medical insurance during the study period. Baseline comparisons did not show significant differences between CTC-tested and non-tested patients, but residual selection bias cannot be excluded. Fourth, the NACT subgroup was relatively small and did not include SD or PD cases. Response was recorded as CR or PR rather than uniformly documented pCR or residual cancer burden, limiting the ability to evaluate TK1 as a response-discriminating marker. Finally, the study size was determined by consecutive eligible cases during the prespecified study period, and no formal a priori sample-size calculation was performed.

These limitations indicate that the present findings should be considered hypothesis-generating. Larger prospective studies should use standardized subtype classification, assay-specific TK1 cutoffs, predefined serial blood sampling schedules, standardized pathological response endpoints, and long-term follow-up. Only such studies can determine whether TK1 adds clinically meaningful information beyond established pathological and molecular risk factors.

## 5. Conclusion

In this cohort, serum TK1 was associated with younger age, LNM, higher histological grade, Ki-67 expression, and the HER2-positive/TNBC high-risk phenotype after adjustment. TK1 also decreased markedly after NACT in both CR and PR groups. These findings support serum TK1 as a proliferation-related marker associated with high-risk BC features and treatment-related biological change. Further prospective studies with standardized response assessment and long-term outcome data are needed before TK1 can be considered a validated marker of long-term outcome or used to guide treatment decisions.

## Author contributions

**Data curation:** Yanze Liu, Jiaqi Liu.

**Supervision:** Jiaqi Liu.

**Writing – original draft:** Yanze Liu, Jiaqi Liu.

**Writing – review & editing:** Jiaqi Liu.
